# A united model for diagnosing pulmonary tuberculosis with random forest and artificial neural network

**DOI:** 10.3389/fgene.2023.1094099

**Published:** 2023-03-09

**Authors:** Qingqing Zhu, Jie Liu

**Affiliations:** Anhui Provincial Tuberculosis Institute, Hefei, Anhui, China

**Keywords:** pulmonary tuberculosis, diagnosis, biomarker, RF, ANN

## Abstract

**Background:** Pulmonary tuberculosis (PTB) is a chronic infectious disease and is the most common type of TB. Although the sputum smear test is a gold standard for diagnosing PTB, the method has numerous limitations, including low sensitivity, low specificity, and insufficient samples.

**Methods:** The present study aimed to identify specific biomarkers of PTB and construct a model for diagnosing PTB by combining random forest (RF) and artificial neural network (ANN) algorithms. Two publicly available cohorts of TB, namely, the GSE83456 (training) and GSE42834 (validation) cohorts, were retrieved from the Gene Expression Omnibus (GEO) database. A total of 45 and 61 differentially expressed genes (DEGs) were identified between the PTB and control samples, respectively, by screening the GSE83456 cohort. An RF classifier was used for identifying specific biomarkers, following which an ANN-based classification model was constructed for identifying PTB samples. The accuracy of the ANN model was validated using the receiver operating characteristic (ROC) curve. The proportion of 22 types of immunocytes in the PTB samples was measured using the CIBERSORT algorithm, and the correlations between the immunocytes were determined.

**Results:** Differential analysis revealed that 11 and 22 DEGs were upregulated and downregulated, respectively, and 11 biomarkers specific to PTB were identified by the RF classifier. The weights of these biomarkers were determined and an ANN-based classification model was subsequently constructed. The model exhibited outstanding performance, as revealed by the area under the curve (AUC), which was 1.000 for the training cohort. The AUC of the validation cohort was 0.946, which further confirmed the accuracy of the model.

**Conclusion:** Altogether, the present study successfully identified specific genetic biomarkers of PTB and constructed a highly accurate model for the diagnosis of PTB based on blood samples. The model developed herein can serve as a reliable reference for the early detection of PTB and provide novel perspectives into the pathogenesis of PTB.

## Introduction

Tuberculosis (TB) affects nearly five million adult males, 3.5 million adult females, and 1 million children, and there are approximately 10.4 million cases of TB worldwide (Jeremiah et al., 2022). Owing to the increasing global population, public health departments are continually aiming to improve the diagnostic efficiency of TB and reduce its rate of transmission. Microscopic examination of sputum smears for acid-fast bacilli and sputum cultures are commonly used for diagnosing pulmonary TB (PTB) worldwide. However, these microbiology-based approaches and culture methods are time consuming, and the probability of infection is high (Barac et al., 2019). It is therefore urgently necessary to study and development non-sputum-based, simple, sensitive, and specific tests for diagnosing PTB. The biomarkers of PTB have been increasingly explored in the last three years owing to several studies on the identification of novel diagnostic biomarkers and development of novel diagnostic methods for PTB (Khambati et al., 2021; Morrison and Mcshane, 2021; Khimova et al., 2022). These studies have paved the way for the diagnosis and identification of novel biomarkers of PTB. While there has been success in clinical use of pathogen-based biomarkers in the form of Cepheid GeneXpert and Urine Lipoarabinomannan (LAM), host-based biomarkers are in less advanced stages of development (Nogueira et al., 2022). Based on previous literature, the present study aimed to identify more specific biomarkers of PTB using blood samples.

Blood-based gene expression signatures are the most potential biomarkers for diagnosing PTB. According to the target product profile (TPP) for non-sputum biomarker triage tests published by the World Health Organization in April 2014, TPPs require a minimum diagnostic sensitivity of 90% and specificity of 87% for the diagnosis of PTB in adults (Denkinger et al., 2019). Several recent studies have demonstrated that whole-blood RNA signatures can be used for predicting active TB infections and determining the progression of *Mycobacterium tuberculosis* infections in individuals who are at a risk of developing active TB (Kaforou et al., 2013; Blankley et al., 2016; Sweeney et al., 2016; Zak et al., 2016).

The increasing use of high-throughput sequencing technologies in the last decade has enabled the investigation of various aspects of diverse diseases (Dillies et al., 2013; Sullivan et al., 2017). Large volumes of high-throughput data have been stored in public platforms owing to the rapid development of high-throughput sequencing technology. These data can therefore be used for selecting critical indicators or feature biomarkers, which is a significant challenge for the development of diagnostic models. Machine learning techniques, including random forest (RF) and artificial neural network (ANN), can provide novel insights for solving this problem, and have been widely employed in previous studies for constructing diagnostic models by analyzing sequencing data (Dillies et al., 2013; Sullivan et al., 2017). Random Forest algorithm can perform random sampling to screen the target biomarkers and has high predicted accuracy (Byeon, 2019). Furthermore, the Artificial Neural Network can be used to evaluate the weight of target biomarkers screened by RF and construct the predicted model for PTB with divided training and validation datasets (Curchoe et al., 2020). However, multi-biomarker-based diagnostic models and the combination of RF and ANN have not been employed for the diagnosis of TB to date.

**FIGURE 1:**
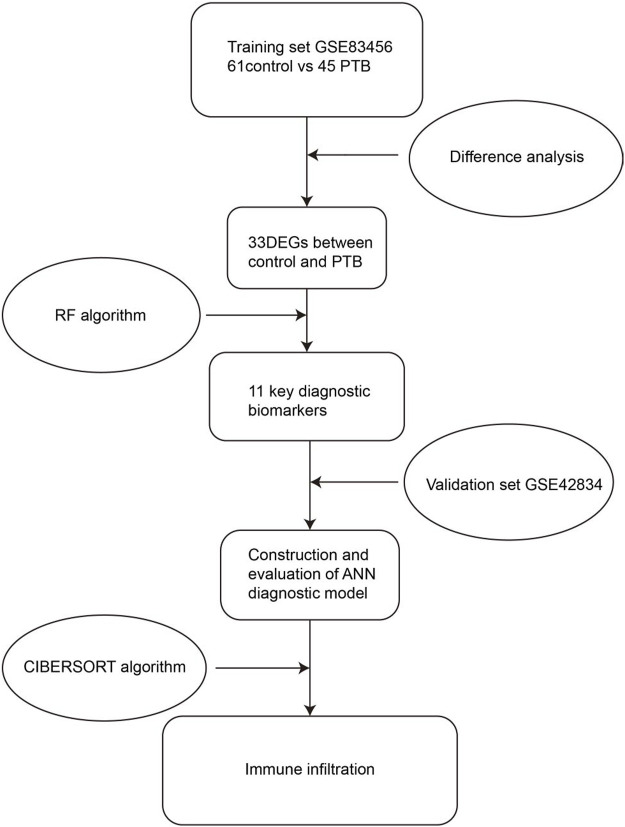
Flow chart of the present study. DEGs, differentially expressed genes; RF, random forest; ANN, artificial neural network.

The present study aimed to construct a multi-mRNA diagnostic model for the diagnosis of PTB. To this end, the genes that were differentially expressed between the PTB and control samples in the public datasets in the Gene Expression Omnibus (GEO) database were initially identified. The essential biomarkers for classifying PTB were screened using an RF classifier, and the weight of each biomarker was determined using ANN. A diagnostic model was subsequently developed based on these biomarkers and the accuracy of the model in discriminating between PTB and control samples was verified by receiver operating characteristic (ROC) curve analysis. The area under the curve (AUC) of the training (GSE83456) and validation (GSE42834) cohorts was determined to be 1.000 and 0.946, respectively. The high accuracy indicated that the diagnostic model constructed herein met the necessary requirements for the clinical diagnosis of PTB. The protocol and algorithms used in the present study are depicted in Figure 1.

## Methods

### Data processing

In this study, two RNA expression datasets were initially retrieved from the GEO database using the keywords “tuberculosis, normal.” The GSE83456 and GSE42834 datasets were processed using the GPL10558 platform of an Illumina HumanHT-12 V4.0 Expression BeadChip system. Based on the available literature on the use of machine learning for the diagnosis of diseases, we assumed that the sample size of the two datasets was appropriate for developing a machine learning-based diagnostic model. The obtained RNA-seq data were subsequently annotated and normalized using R software (version 4.2.1). The GSE83456 and GSE42834 datasets were selected as the training and validation cohorts, respectively.

### Identification of differentially expressed genes (DEGs)

The DEGs between the PTB and control samples in the training set were identified using the limma package in R, with *p* < 0.05 and |log2foldchange (FC)| >1.0. The DEGs were visualized using the pheatmap and ggplot2 packages in R.

### Functional enrichment analysis

The identified DEGs were subjected to Gene Ontology (GO) enrichment analysis for investigating the biological functions of the DEGs, using the clusterProfiler package in R (version 4.1.5). GO terms with *p* < 0.05 were considered to be significantly enriched. The Metascape webserver (http://metascape.org) was also used to annotate the enriched biological pathways for comprehensive analysis of the biomarkers. The most enriched functions or pathways were subsequently displayed using bubble and bar plots.

### Screening significantly enriched DEGs using RF

The DEGs were further screened using the randomForest package in R software. The optimal tree number was first identified based on the best stability and lowest error rate by calculating the error rate of each of the 1–500 trees. We established an RF model based on the optimal tree number for screening the specific PTB genes as candidate biomarkers using the mean decrease in Gini coefficient. In the RF algorithm, a gene importance value greater than 2 is considered to be a common screening criterion, and has been used in other studies on machine learning-based diagnostic models.

### Construction and evaluation of an ANN-based diagnostic model

In order to construct an ANN-based diagnostic model, the min-max method was used for normalizing the input data, which were subsequently converted into the “Gene Score” according to the gene expression levels. For instance, the expression of an upregulated gene was denoted as 1 if the expression level was higher than the median expression value across all the samples, or denoted as 0 in other instances. Similarly, the expression of a downregulated gene was generally denoted as 0, or as 1 if the expression level was higher. A neural network-based classification model was subsequently constructed by calculating the weights of the significantly enriched DEGs using the neuralnet package in R (version 4.2.1). A neural network contains an input layer, a hidden layer, and an output layer. In this study, the number of hidden layers was set to 5, and the number of output parameters was set to 2 nodes (contract/segment). Additionally, the AUC value of the training cohort was calculated using the pROC package in R (version 4.2.1). The accuracy of the model was also verified using the independent GSE42834 cohort.

### Analysis of immune infiltration

CIBERSORT is a deconvolution algorithm that is used for quantifying cell types based on the gene expression profiles, and was used to determine the abundance of 22 types of immune cells in the PTB and control tissues. Using the CIBERSORT algorithm, the immune infiltration landscape in the GSE83456 cohort was comprehensively analyzed, and the differences between the control and PTB groups were depicted using waterfall and correlation plots.

### Statistical analyses

The differences in gene expression between the control and PTB samples were compared using Student’s t-tests. The categorization effects of the critical biomarkers on the PTB and control specimens were determined using ROC curves and the AUC using the pROC package in R. Statistical analysis was performed using the R software (version 4.2.1) and GraphPad Prism (GraphPad Prism, USA). *p* < 0.05 was considered to be statistically significant, unless otherwise stated.

## Results

### Data processing and identification of DEGs

**FIGURE 2:**
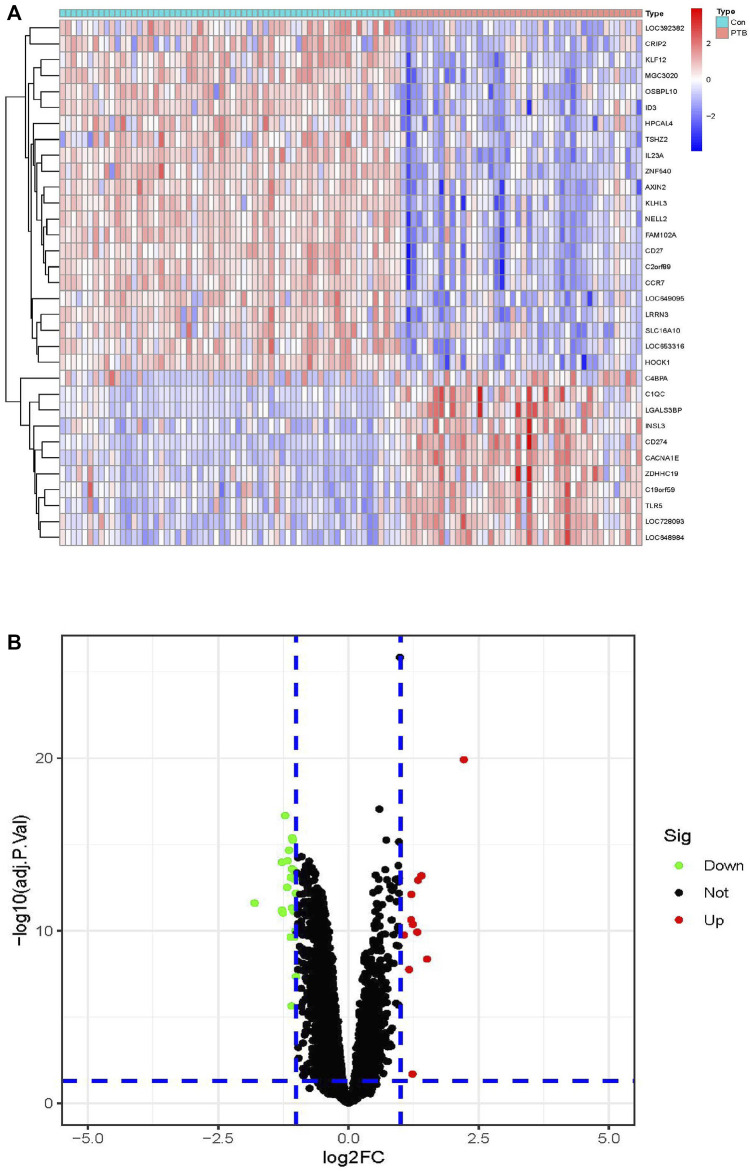
Identification of DEGs in the training cohort. **(A)** The heatmap of the 33 DEGs, including 11 upregulated and 22 downregulated ones. PTB were represented by red samples, normal were represented by blue samples. Red blocks indicate high-expressed genes, and blue blocks indicate low-expressed genes. Con, control group; PTB, Pulmonary Tuberculosis. **(B)** Volcano plots of all DEGs in the GSE83456 dataset. Two dotted lines on the *X*-axis represent the value of log2FC is −1 and 1. The dotted line on the *Y*-axis represent the adj.*p*.value is 0.05. Red dots represent high-expressed genes, blue dots represent low-expressed genes and black dots represent not significant changed genes.

The limma package in R was used for identifying the DEGs between the 45 PTB and 61 control samples using the classical Bayesian algorithm, based on the following criteria: *p* < 0.05 and |log2FC| >1. A total of 33 DEGs were finally identified, including 11 and 22 DEGs that were significantly upregulated and downregulated, respectively. As depicted in Figure 2A, the expression of these DEGs differed significantly between the PTB and control groups. The results were graphically represented using a volcano plot, which further revealed the differences in gene expression and statistical significance of the DEGs (Figure 2B).

### Functional enrichment analysis of DEGs

**FIGURE 3:**
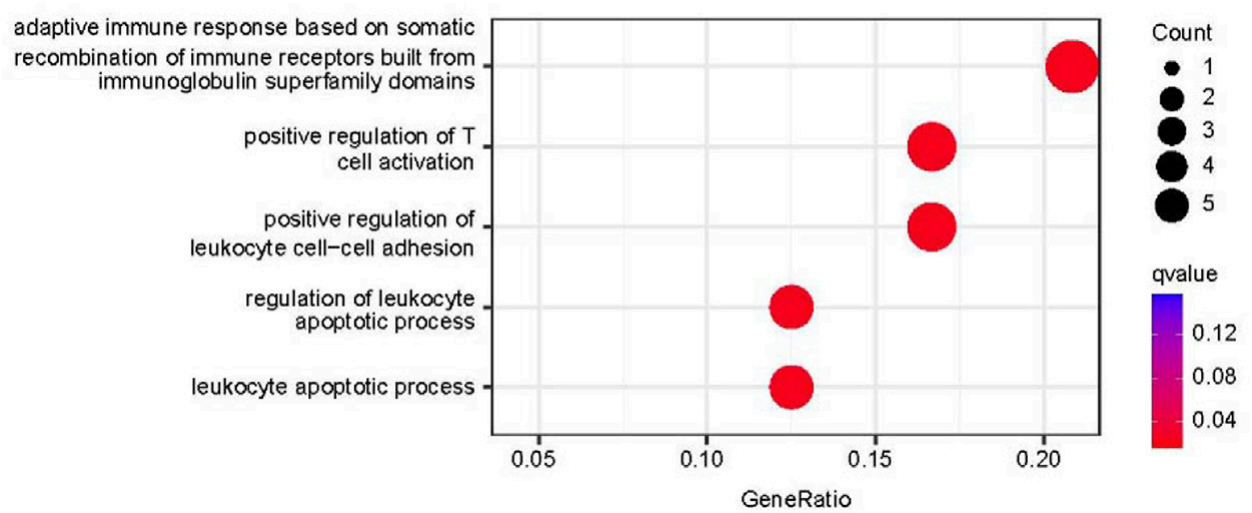
Functional enrichment analysis results. Top five enriched GO terms in biological process (BP).

**FIGURE 4:**
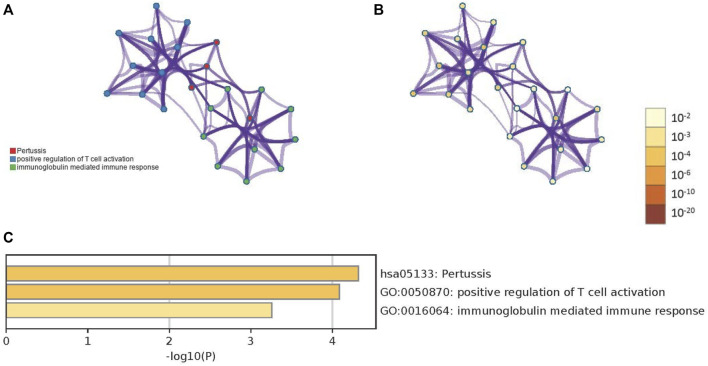
The results of Metascape analysis. **(A)** The network of enriched terms. The 3 clusters were identified and rendered network graphics, in which terms with a similarity score > 0.3 are connected by an edge. The thickness of the edge represents the similarity score. **(B)** Colored by *p*-value, terms containing more genes tend to have a more significant *p*-value **(C)** Bar graph of enriched terms. Values of *p* determine the color of the bar. The values of *p* are lower, and the color is more profound.

The biological significance of the 33 DEGs in the pathogenesis of PTB was investigated by GO pathway enrichment analysis using the clusterProfiler package in R. The findings revealed that the 33 DEGs were primarily involved in immune-related functions, including adaptive immune response based on somatic recombination of immune receptors comprising immunoglobulin superfamily domains, positive regulation of T cell activation, positive regulation of leukocyte cell-cell adhesion, regulation of leukocyte apoptotic process, and leukocyte apoptotic process. The findings are presented in a bubble plot (Figure 3). The Metascape webserver was also used for annotating the enriched GO terms. The results of Metascape analysis revealed that the three pathways of DEGs were significantly enriched (Figure 4A, B and C).

### Screening key genes using an RF classifier

**FIGURE 5:**
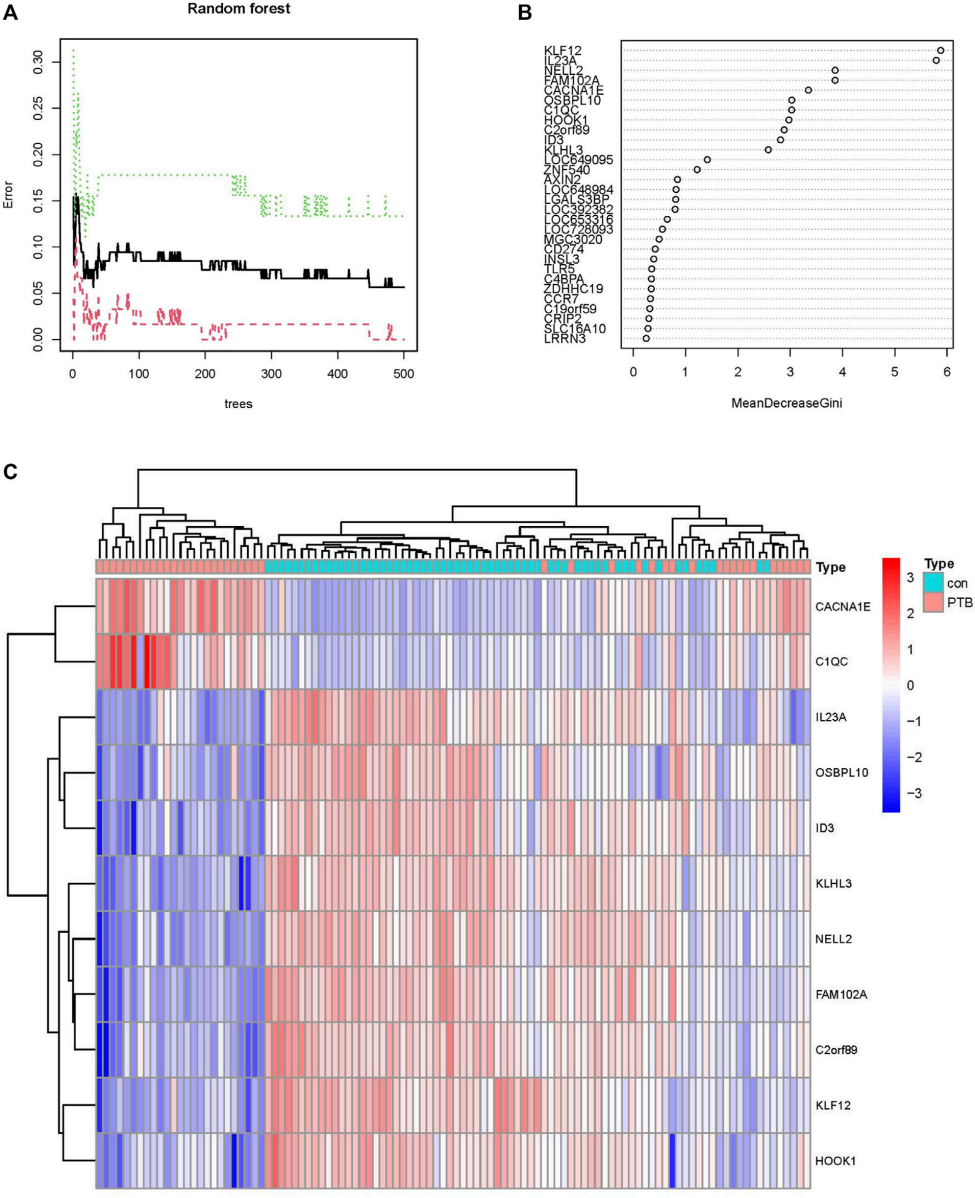
Screening PTB biomarkers by random forest. **(A)** The relations between the error rate and the number of decision trees. **(B)** The Gini coefficient method in random forest modeling of the train cohort. The genetic variable is on the *y*-axis and the importance index is on the *x*-axis. **(C)** Heatmap of the 11 specific periodontitis biomarkers.

In order to identify the reliable diagnostic biomarkers of PTB, the DEGs were classified using an RF classifier. According to Figure 5A, which depicts the relationship between the RF tree number and the error rate of the model, the trees with the lowest error rate ntrees value (ntrees = 31) were selected. Based on the model accuracy and decreased mean square error, the Gini coefficient method was used for measuring the importance of all the variables. The results of MeanDecreaseGini are provided in Figure 5B. Kruppel-like factor 12 (KLF12) was identified as the most important biomarker. A set of 11 specific biomarkers, including KLF12, interleukin 23 subunit alpha (IL23A), neural EGFL-like 2 (NELL2), Family With Sequence Similarity 102 Member A (FAM102A), Calcium Voltage-Gated Channel Subunit Alpha1 E (CACNA1E), Oxysterol Binding Protein like 10 (OSBPL10), complement component C1q (C1QC), Hook Microtubule Tethering Protein 1 (HOOK1), Chromosome 2 open reading frame 89 (C2orf89), inhibitor of DNA binding 3 (ID3), and Kelch Like Family Member 3 (KLHL3), with significance >2 were selected as critical biomarkers for further analysis. The heatmap revealed that CACNA1E and C1QC were upregulated in the PTB group, while the remaining 9 genes were downregulated (Figure 5C).

### Construction of the ANN model

**FIGURE 6:**
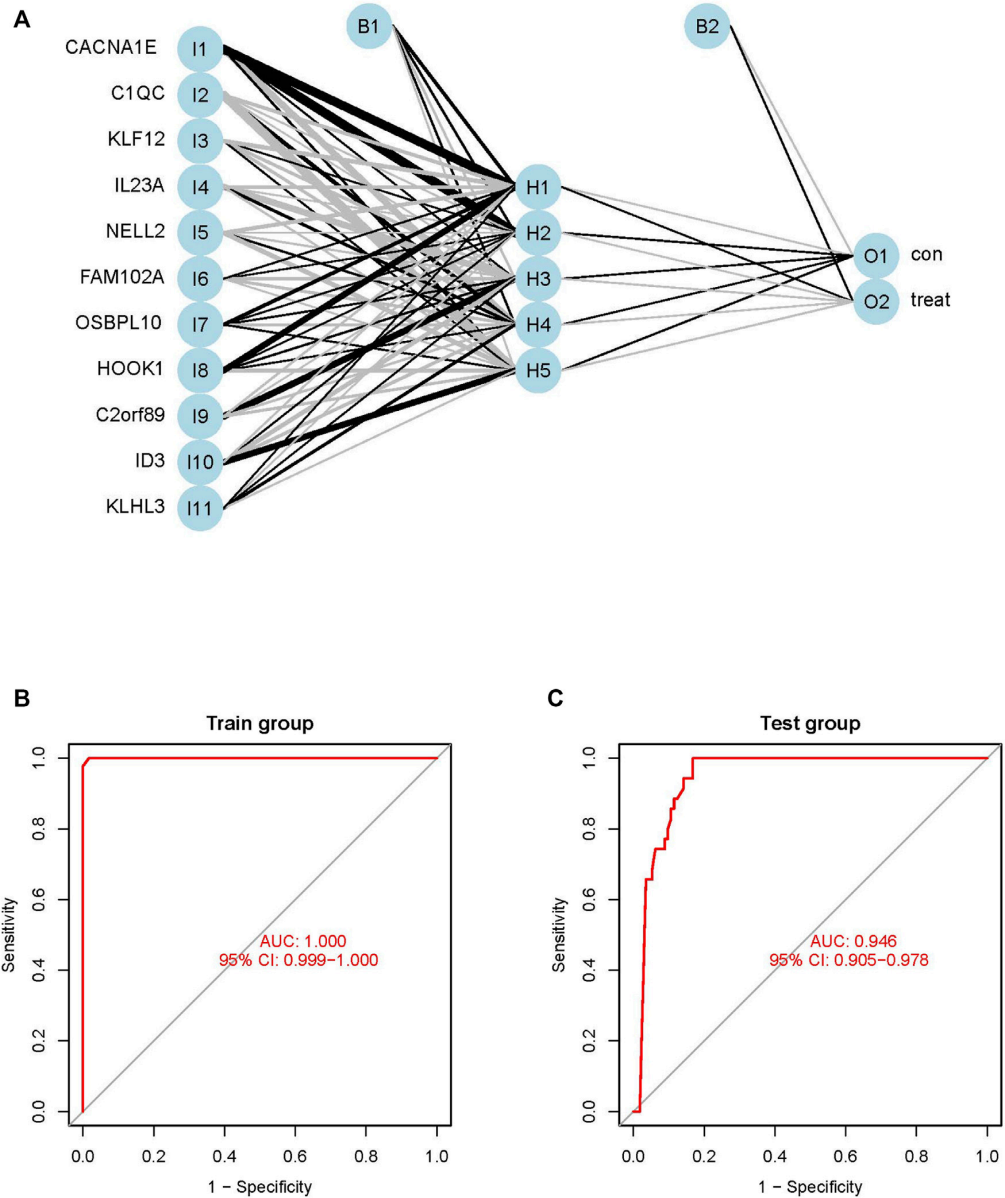
Construction and evaluation of ANN diagnostic model. **(A)** Topology, which include one input layer, one hidden layer and one output layer, the visualization of the artificial neural network. **(B)** ROC curves of train model in the GSE83456 dataset. **(C)** ROC curves of test model in the GSE42834 dataset.

The weights of each of the biomarkers are provided in Supplementary Table S1. The weights of the 11 biomarkers were analyzed using ANN, based on the gene scores. The ANN model consisted of one input layer, one hidden layer, and one output layer, as depicted in Figure 6A. The input layer included 11 neurons, hidden layer included five neurons and output layer included 2 neurons. The absolute partial derivative of the error function was less than 0.01.

### Validation of the ANN model

The performance of the ANN model was determined using the pROC package in R, and the AUC of the training GSE83456 cohort was 1.000. This indicated that the ANN model performed exceptionally well in diagnosing PTB (Figure 6B). The ANN model also demonstrated outstanding performance with the independent GSE42834 validation cohort, and the AUC of the validation cohort was determined to be 0.946 (Figure 6C).

### Assessment of immune infiltration

**FIGURE 7:**
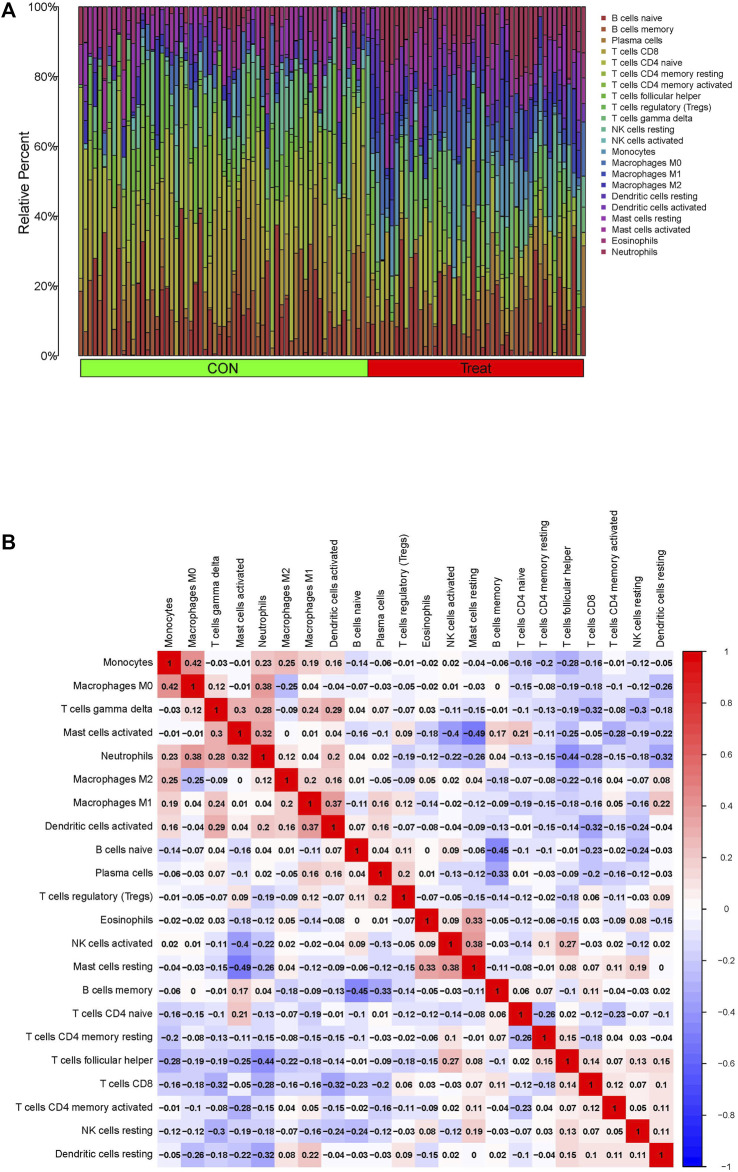
Immune infiltration assessment *via* the CIBERSORT in the GSE83456 dataset. **(A)** Composition of 22 immunocytes on PTB samples and normal. **(B)** The relationship among 22 immunocytes are displayed in correlation matrix.

The present study further investigated the correlation between the ratios of the 22 types of immunocytes in the PTB and control specimens using the CIBERSORT algorithm. The composition of the immunocytes in the PTB and normal samples and the relationships among the immunocytes are provided in Figure 7A. The findings revealed a positive correlation between the levels of M0 macrophages and monocytes, and between the levels of M0 macrophages and neutrophils. However, there was a negative correlation between the abundance of resting mast cells and activated mast cells, levels of memory B cells and naïve B cells, and the ratio of follicular helper T cells and neutrophils (Figure 7B).

## Discussion

The early detection and diagnosis of PTB can reduce its chances of transmission; therefore, identifying specific biomarkers for the prediction of PTB is crucial for controlling disease progression. RF and ANN can be combined for developing reliable diagnostic models for certain diseases, including osteoarthritis and hypertrophic cardiomyopathy (Xie et al., 2020; Li S et al., 2022; Li Z B et al., 2022). RF and ANN are advanced tools for diagnosing PTB, but their main limitation is the necessity for trained and qualified personnel for implementing these tools, as the construction of neural networks, which includes training and testing, is a challenging task. Additionally, the use of statistical tools for diagnosing diseases continues to be a matter of difficulty.

The present study identified 33 DEGs between PTB and control samples in the GSE83456 cohort. A total of 11 candidate genes were identified using an RF classifier, and an ANN algorithm was used for computing the weights of these genes. A classification model was constructed for the diagnosis of PTB, and a ROC curve was generated for assessing the efficacy of the classification by the ANN model. An independent GSE42834 cohort was used for determining the reliability of the classification model.

The results of enrichment analysis demonstrated that the majority of DEGs were primarily enriched in immune-related functions. It has been reported that T cells are involved in the development of TB, and the activation of T cells enhance resistance to *M. tuberculosis* infections (Feruglio et al., 2017). Leukocytes are also implicated in the inflammatory pathogenesis of TB (Ocana-Guzman et al., 2021). However, adaptive immune responses based on somatic recombination of immune receptors comprising immunoglobulin superfamily domains have not been previously reported in TB, and may serve as a novel therapeutic target for PTB. Altogether, the findings revealed that these DEG identified herein are positively involved in the immune processes in PTB.

Of the 11 genes screened using the RF classifier, KLF12 (Natarajan et al., 2022), IL23A (Khader et al., 2011), NELL2 (Yang et al., 2015), OSBPL10 (Li et al., 2022), C1QC (Cai et al., 2014), and ID3 (Han et al., 2021) have been identified as candidate biomarkers of TB in previous studies.

Notably, the present study identified additional five genes that have not been previously shown to be associated with the pathogenesis of PTB. The KLHL3 gene, which is downregulated in PTB, encode proteins that are components of the CullinRING E3 ubiquitin ligase complex and are involved in the ubiquitin-proteasome system. The complex degrades proteins and also plays an essential role in maintaining cellular functions (Zhang et al., 2022). It has been reported that the ubiquitin-proteasome system also plays a role in inducing CD8^+^ T cells (Shen et al., 2008). Therefore, the downregulation of KLHL3 may suppress the degradation of proteins that regulate the ubiquitin-proteasome system and subsequently induce CD8^+^ T cells that participate in the pathogenesis of PTB.

The present study revealed that the expression of HOOK1 is downregulated in PTB. A previous study reported that enhancing the interaction between HOOK1 and CD147 may increase the exosomal levels of amyloid-β (Xie et al., 2018). The deposition of amyloid-β has been reported to be associated with tuberculous meningitis (Stroffolini et al., 2021). We therefore speculated that HOOK1 may affect the deposition of amyloid-β to regulate the pathogenesis of PTB. CD147++ Tregs cells, a recently described highly suppressive and activated subset of human Tregs, are capable of producing proinflammatory cytokines in TB (Feruglio et al., 2015). These studies collectively suggest that HOOK1 may participate in the pathological processes of PTB *via* multiple pathways.

The CACNA1E protein can mediate the entry of calcium ions into excitable cells and regulate various calcium-dependent processes. Numerous studies have reported that calcium channel blockers have anti-tuberculosis potential (Lee et al., 2015; Song et al., 2015; Lee et al., 2021). Therefore, the upregulation of CACNA1E in PTB may result in the activation of calcium channels and lead to the pathogenesis of PTB.

The present study is the first to identify the association between FAM102A and the pathogenesis of TB. The findings revealed that the expression of FAM102A was downregulated in the samples of PTB in this study. Notably, protein-protein interaction (PPI) analysis with STRING (string-db.org) revealed that the FAM102A protein interacts with NELL2, which has been confirmed as a biomarker of TB. It has been additionally reported that NELL2 plays a crucial role in protecting cells from environments that induce cell death (Kim et al., 2015). The deficiency of NELL2 induces mitochondria-dependent cellular apoptosis and inhibits cellular proliferation by phosphorylating and activating extracellular signal-regulated kinase 1/2 (ERK1/2) (Liu et al., 2021). These findings suggest that FAM102A can function as a biomarker of PTB by interacting with NELL2, and subsequently influence cellular apoptosis and regulate the pathogenesis of PTB.

The C2orf89 protein, also referred to as TRABD2A, could be involved in activating resting CD4^+^ T cells but not activated CD4^+^ T cells. The TRABD2A protein is located on the plasma membrane of resting CD4^+^ T cells and disappears following the activation of T cells (Liang et al., 2019). CD4^+^ T cells produce cytokines, which are vital in controlling *M. tuberculosis* infections (Ferreira et al., 2021). It is therefore likely that the production of cytokines, including interferon (IFN)-γ, by activated CD4^+^ T cells suppresses *M. tuberculosis* infections and downregulates the TRABD2A protein located on the plasma membrane of resting CD4^+^ T cells.

The particularities of our research are combining RF and ANN methods innovatively, and multiple biomarkers combined diagnosis, which showed outstanding results in the predictive power aspect. The AUC of train model and valid model are both greater than 0.9. Compared with several literatures (Manisha Singh et al., 2022; Yu Dong Zhang, 2020) which utilize the chest radiography images to detect Pulmonary Tuberculosis with the help of machine learning tools (CAD, DL, ICNN), our work is analysing biomarkers from peripheral blood biomarkers and constructing diagnostic model for PTB with the combination of RF and ANN. Although, RF, ANN, or other machine learning had been utilized in diagnosing TB (Dande and Samant, 2018; Orjuela-Canon et al., 2022), combining RF and ANN to diagnose PTB had never been reported. Our samples are both from human blood, we could design the diagnostic kit based on the eleven biomarkers and to detect the blood which sampling from human fingers. It is we choose figure blood sampling rather than sputum smear and X-ray that bring us the diagnostic convenience and safety. However, the present study has certain limitations. Firstly, although our diagnostic model performed well, the number of samples in the training and validation datasets was relatively small. Therefore, independent patient cohorts with a larger sample size are necessary for evaluating the performance of the ANN-based classification model developed herein, and sufficient samples need to be collected from affiliated hospitals for this purpose. Secondly, all the samples were only classified as normal or PTB, which may influence the results of screening; therefore more subtypes of PTB should be considered in future studies. Thirdly, the correlation between the novel biomarkers and the pathogenesis of PTB remain to be determined, and further experimental studies are necessary for elucidating the underlying mechanisms by which the biomarkers regulate the pathogenesis of PTB. Altogether, the model developed herein has high accuracy and excellent diagnostic convenience owing to the use of data obtained from routine blood tests.

## Conclusion

Altogether, the present study successfully constructed a novel diagnostic model for PTB. As the diagnostic method is based on peripheral blood tests, a diagnostic kit can be designed based on the 11 biomarkers identified herein, which is highly convenient for the rapid and accurate diagnosis of PTB. The diagnostic model, biomarkers, and the peripheral blood test method discussed herein provide novel insights into the underlying mechanisms and can aid further studies on the clinical diagnosis of PTB. However, further experimental studies are necessary for determining the underlying mechanisms by which the identified biomarkers regulate the pathogenesis of PTB.

## Data Availability

The original contributions presented in the study are included in the article/Supplementary Material, further inquiries can be directed to the corresponding author.
